# Serum Sickness-Like Reaction: Drug-Induced Cutaneous Disease in a Child

**DOI:** 10.5826/dpc.1103a29

**Published:** 2021-07-08

**Authors:** Ana Carolina Galvão dos Santos de Araujo, Liana Moura de Almeida, Ana Paula Moura de Almeida, Elisa Fontenelle de Oliveira, Mayra Carrijo Rochael, Rodrigo Aires de Morais, Hudson Dutra Rezende

**Affiliations:** 1 Álvaro Alvim School Hospital. Department of Dermatology. Campos dos Goytacazes, Rio de Janeiro, Brazil; 2 Fernandes Figueira Institute/FIOCRUZ. Department of Pediatric Dermatology. Rio de Janeiro, Rio de Janeiro, Brazil; 3 Fluminense Federal University. Department of Pathology. Niterói, Rio de Janeiro, Brazil; 4 Campos dos Goytacazes, Rio de Janeiro, Brazil

**Keywords:** Serum Sickness, Drug Hypersensitivity, Allergic Reaction, Anti-Bacterial Agents, Clavulanic Acid

## Introduction

Serum sickness is defined as a type III hypersensitivity reaction, which leads to complement activation and hypocomplementemia, small vessel vasculitis, and nephropathy. From a clinical perspective it resembles Serum Sickness-Like Reaction (SSLR), presenting clinical similar findings. These are both rare diseases that can manifest with urticarial rash, fever, and arthralgia, mainly differing by their etiopathology and laboratory findings [1,2]. SSLR pathogenesis remains unclear but is probably related to an inflammatory response to drug metabolites, more commonly, following beta-lactams use. It is not mediated by immunocomplexes and does not manifest hypocomplementemia [1,2] .

## Case Report

**Figure 1:**
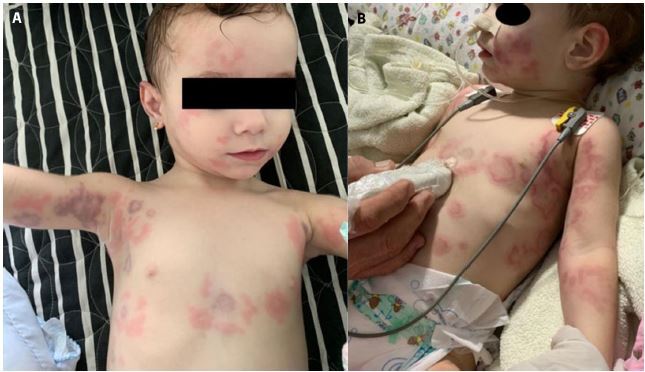
(A)Diffuse erythematous and violaceous, edematous plaques on trunk, axillary region, and face. (B) After 2 days, appearance internal violaceous halo.

**Figure 2:**
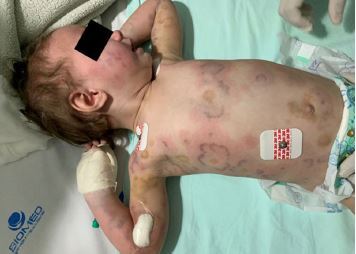
Diffuse arcuate and annular pink edematous plaques with central purple annular patches surrounding a yellowish center.

An 18-month-old girl, presented with spread, non-pruritic, erythematous-edematous plaques (some violaceous) characterized by irregular borders (Figure 1A). 2 days after, a violaceous inner halo appeared on the plaques and the patient developed systemic symptoms, including joint edema, arthralgia, limited mobility, irritability, hyporexia, and fever (Figure 1B and 2). The patient had previously received oral amoxicillin/clavulanate and dipyrone treatment 5 days before the skin lesions appeared, due to cough and fever. Oral prednisone 0,5mg/kg and hydroxyzine did not help fade the lesions and following further clinical deterioration the situation worsened until she was admitted at the Intensive Care Unit (ICU).

On the first day of hospitalization leukocytosis was at 24.730/mm^3^, after 2 days it raised to 31.800/mm^3^; C reactive protein analysis increased from 29,9mg/dL on the first day, to 90,2 mg/dL. Liver enzymes and renal function were normal. Transthoracic echocardiogram did not demonstrate any abnormality.

**Figure 3:**
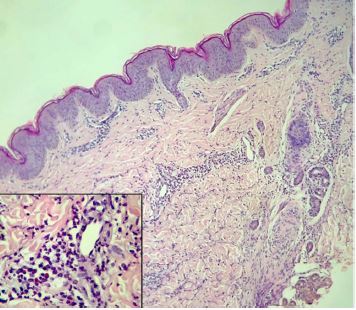
(H&E, ×100) (magnification ×400) Normal epidermis, and perivascular, periadnexal and interstitial cell infiltrate inflammatory infiltrate, consisting of lymphocytes, neutrophils and eosinophils, in addition to edema without vasculitis.

A skin biopsy was performed, and the histopathology showed normal epidermis, perivascular, periadnexal, and interstitial inflammatory infiltrate, consisting of lymphocytes, neutrophils and numerous eosinophils, in addition to edema, without vasculitis (Figure 3). Antibiotic treatment was suspended, antipyretic and oral corticosteroids (0,5 mg/kg/day) were maintained, in addition to venous hydration. After 3 days of hospitalization, she was discharged with significant improvement.

## Conclusions

Diagnosis of SSLR is clinical and can be made when annular, erythematous edematous plaques, similar to an urticarial reaction, usually turning violet, giving the lesion an ecchymotic aspect, appear combined with systemic symptoms, such as arthralgia/arthritis, lymphadenopathy, malaise, and fever, following 8 to 14 days of drug exposure. SSLR diagnosis, needs however to be distinguished from other, potentially more serious conditions such as Lyme disease, endocarditis, erythema multiforme, Kawasaki’s disease, and in some rare situations to neoplastic diseases. In this case, one of the first hypotheses considered before the dermatology evaluation, was meningococcemia, and the patient almost underwent a lumbar puncture.

The treatment involves the discontinuation of the culprit drug and, if necessary, the use of corticosteroids, anti-inflammatory, antihistamines, or sometimes, intravenous gamma globulin administration [1,2]. Early recognition of this condition leads to less family anxiety and a non-iatrogenic management, avoiding unnecessary and potentially harmful prescriptions [1,2].
